# Cooperating transcription factors mediate the function of estrogen receptor

**DOI:** 10.1007/s00412-012-0392-7

**Published:** 2012-11-29

**Authors:** Elisa Fiorito, Madhumohan R. Katika, Antoni Hurtado

**Affiliations:** 1 Breast Cancer Research group, Centre for Molecular Medicine Norway, Nordic EMBL Partnership, University of Oslo and Oslo University Hospital, 0318 Oslo, Norway; 2 Department of Genetics, Institute for Cancer Research, Oslo University Hospital, 0424 Oslo, Norway

## Abstract

Estrogen receptor (ER) is a hormone-regulated transcription factor that controls cell division and differentiation in the ovary, breast, and uterus. The expression of ER is a common feature of the majority of breast cancers, which is used as a therapeutic target. Recent genetic studies have shown that ER binding occurs in regions distant to the promoters of estrogen target genes. These studies have also demonstrated that ER binding is accompanied with the binding of other transcription factors, which regulate the function of ER and response to anti-estrogen therapies. In this review, we explain how these factors influence the interaction of ER to chromatin and their cooperation for ER transcriptional activity. Moreover, we describe how the expression of these factors dictates the response to anti-estrogen therapies. Finally, we discuss how cytoplasmatic signaling pathways may modulate the function of ER and its cooperating transcription factors.

## Introduction

The steroid hormone estrogen and the estrogen receptor alpha (ER) are necessary for the physiology of the female reproductive system (Musgrove and Sutherland 2009). These factors play an essential role in the breast, ovaries, and uterus, where they control cell division and differentiation, and the deregulation of ER transcriptional activity may result in an increased proliferation and eventually in cancer onset.

Breast cancer is a heterogeneous disease with a different subgroup of patients showing distinct molecular profiles (Perou et al. 2000; Sorlie et al. 2001; Curtis et al. 2012; Gray and Druker 2012). However, the most widespread type is the luminal group of tumors, and they share the common feature of being positive for the expression of ER (Dowsett 2001; Prat and Baselga 2008). ER is a transcription factor that mediates the response to estrogens and to anticancer therapies, including the selective estrogen receptor modulator (SERM) tamoxifen (Katzenellenbogen and Frasor 2004; Hurtado et al. 2011). Our knowledge of how ER elicits transcription has increased significantly during the last years. The incorporation of new technologies such as high-throughput sequencing has been crucial for a deep understanding of ER function. Chromatin immunoprecipitation (ChIP) combined with sequencing studies in breast cancer cell lines and human tissue shows a dispersed occupancy pattern of ER binding sites bearing heterogeneous recognition motifs (Carroll et al. 2006; Lin et al. 2007; Ross-Innes et al. 2012). Estrogen and tamoxifen can affect the gene expression profile by inducing thousands of ER binding events (Frasor et al. 2006; Hurtado et al. 2011). Moreover, ER binds to chromatin with a multitude of transcription factors (ER-cooperating factors) that influence transcriptional activity of ER and ultimately affect the outcome of anti-estrogen therapies (Carroll et al. 2005; Laganiere et al. 2005a, b; Cheng et al. 2006; Hurtado et al. 2011; Kong et al. 2011).

A second group of breast cancer patients is characterized by an amplification of chromosome region 17q12-21, leading to the overexpression of the epidermal growth factor receptor 2, *ERBB2*/*HER2*/*neu* (Wolff et al. 2007). Moreover, about half of HER2-positive patients are also positive for ER (Dowsett 2001), and the activation of other signaling pathways such as the PI3K pathway is critical for ER/HER-2-positive tumor development (Berns et al. 2007). Yet, the molecular mechanism by which these signaling pathways modulate ER and ER-cooperating factors is not completely understood. In this review, we describe how cooperating factors influence the transcriptional activity of ER, and we speculate how these signaling pathways may modulate the function of ER and ER-cooperating factors.

## Pioneer transcription factors mediate ER binding

**Fig. 1:**
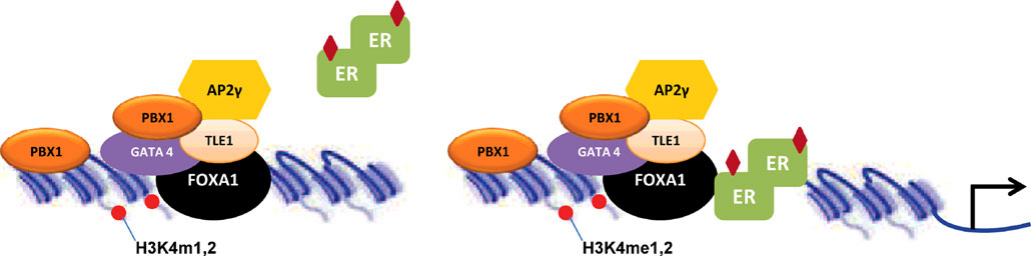
Role of pioneering factors in regulation of ER chromatin interactions. In the absence of pioneering factors, chromatin regions are tightly packed and are not accessible for ER binding. FOXA1, in cooperation with other transcription factors, opens chromatin regions and facilitates ligand–ER binding. PBX1 seems to have a FOXA1-independent effect

ER is a ligand-regulated transcription factor that recognizes a consensus sequence of nucleotides, establishing the binding to DNA, and thereby triggering the recruitment of the transcription machinery. However, most of the genomic regions where ER interacts are in a heterochromatic state (Hurtado et al. 2011), which hinders the interaction of ER with DNA. Pioneer transcription factors interact with chromatin and expose DNA for subsequent transcription factor binding and initiation of transcription (Liu et al. 1991; Monaghan et al. 1993). Genomic analyses of ER binding maps have shown that its union is accompanied with the binding of various transcription factors, which includes Forkhead box A (FOXA) (Carroll et al. 2005; Laganiere et al. 2005a, b; Eeckhoute et al. 2006, 2007), GATA (Krum et al. 2008; Miranda-Carboni et al. 2011), AP2γ (Tan et al. 2011), and PBX1 (Magnani et al. 2011). In this section of the manuscript, we describe their role as pioneer factors (Fig. 1).

FOXA proteins are the most studied pioneer transcription factors that bind to chromatin and enable gene activity. FOXA1 (also known as HNF3α) recruitment to chromatin is mediated by the epigenetic signature consisting of mono- and dimethylated histone H3 on lysine 4 (H3K4me1/me2) (Lupien et al. 2008). The pioneering properties of FOXA1 reside on its protein structure, which contains a winged helix domain that can structurally mimic histone H1 and H5, and thus permits its stable interaction with histone H3 and H4 with high affinity (Cirillo et al. 1998; Kaestner et al. 2000). The high chromatin affinity of FOXA1 is a unique feature that allows its binding to the specific DNA sequences on the nucleosome core and displaces the linker histones, leading to de-compaction of chromatin and facilitation of the binding of other transcription factors. In breast hormone-sensitive and resistant cancer cell lines, almost all ER–chromatin interactions and gene expression changes are dependent on the expression of FOXA1 (Hurtado et al. 2011). Moreover, FOXA1 influences genome-wide chromatin accessibility of ER (Hurtado et al. 2011). Recently, Ross-Innes et al. have established that hormone-resistant breast cancers still recruit ER to the chromatin, and this binding is associated with FOXA1 (Ross-Innes et al. 2012). Interestingly, ER shows a distinct binding profile in patients with poor clinical outcome to anti-estrogen therapies. These newly identified regions are enriched toward the genes that previously were described to predict clinical outcome (Ross-Innes et al. 2012). More recently, Lupien et al. have shown that SNPs associated with breast cancer risk are located in a subset of the FOXA1 binding regions, which influences the binding affinity for the pioneer factor FOXA1 (Cowper-Sal Lari et al. 2012; Zhang et al. 2008). Therefore, data published to date suggest that FOXA1 is a major determinant of estrogen–ER activity in breast cancer.

Six GATA transcription factors have been identified in vertebrates (GATA-1 to GATA-6) (Kouros-Mehr et al. 2008). In breast, GATA-3 is expressed in luminal tumors (Sorlie et al. 2001). However, the mechanism of GATA-3 action or its potential role as a pioneer factor of ER has not been described yet. By contrast, GATA-4 has been shown to have pioneering properties during early development (Bossard and Zaret 1998) and for ER binding in U2OS osteosarcoma cell line (Krum et al. 2008; Miranda-Carboni et al. 2011), which stably expresses exogenous ER and very low levels of FoxA1 (Hurtado et al. 2011). Interestingly, Stender et al. have identified Runx1 as a mediator for ER–DNA interaction in MDA-MB-231 breast cancer cell line (Stender et al. 2010), which stably expresses exogenous ER and is negative for the expression of FOXA1 and HER2. These results support the idea that distinct pioneer proteins influence ER binding in FOXA1-negative tissues.

The Pre-B cell leukemia homeobox 1 factor (PBX1) is a cofactor for homeobox (HOX) transcription factors as it increases their affinity and specificity to chromatin (Moens and Selleri 2006). PBX1 has been described as a pioneer factor whose function is essential for the ER-mediated transcriptional response (Magnani et al. 2011). Magnani et al. demonstrated that estrogen-induced transcriptional response is preferentially associated with regulatory regions where ER co-bounds with PBX1 or PBX1-FOXA1. Moreover, this study also reports a distinct prognostic value for FOXA1 and PBX1. Indeed, the authors point out PBX1, and not FOXA1, as a novel prognostic marker for recurrence in ER-positive breast cancers (Magnani et al. 2011).

Genomic analyses of ER binding sites from ChIP-sequencing experiments also identified enrichment for AP-2 motifs (Tan et al. 2011). The authors demonstrated that perturbations of the expression of the transcription factor AP-2γ prevent ER binding to DNA and gene transcription. Interestingly, the lack of this factor is even affecting ER long-range chromatin interactions, which have been shown to be essential for ER-mediated transcription (Fullwood et al. 2009). Moreover, FOXA1 also occupies the majority of these shared regions. Further molecular studies indicate that both factors collaborate in ER-mediated transcription (Tan et al. 2011).

The Groucho homologue transducin-like enhancer of split 1 (TLE1) is a multitasking transcriptional co-repressor. TLE proteins can associate with condensed chromatin by binding to the histone tails of nucleosomes (Sekiya and Zaret 2007). The Groucho/TLE/Grg family of co-repressors operates in many signaling pathways and distinct biological processes (Jennings et al. 2006), through their association to different partners. For instance, the human homologue of Groucho TLE1 (Stifani et al. 1992) has critical transcription factor partners such as TCF/LEF-1 (Daniels and Weis 2005), hairy/enhancer of split 1 (Dasen et al. 2001; Carvalho et al. 2010), and the AML/CBFa runt domain transcription factor (Levanon et al. 1998). Biologically, the loss of TLE coincides with increased global protein synthesis and enhanced cell proliferation (Ali et al. 2010), which implicates this factor as a general repressor of gene transcription. Moreover, recently, Holmes et al. have published that TLE1 positively assists some ER–chromatin interactions, a role that is distinct from its general role as a transcriptional repressor. The specific silencing of TLE1 inhibits the ability of ER to bind a subset of ER binding sites within the genome, and this is accompanied by perturbations in phospho-RNA Pol II recruitment (Holmes et al. 2012). Interestingly, TLE1 action occurs at regions where FOXA1 binds more weakly (Holmes et al. 2012), suggesting that TLE1 might be more effective in these chromatin regions.

## Function of ER-cooperating factors in hormone-regulated cancers

FOXA1 and GATA3 proteins are expressed in ER-positive luminal breast cancers (Sorlie et al. 2003). In fact, FOXA1 expression is associated with the expression of steroid hormone receptors (ER, progesterone receptor, and androgen receptor) and other variables of good prognosis such as smaller tumor size, lower histological grade, and expression of luminal cytokeratins (CK18 and CK7/8), BRCA1, and E-cadherin (Habashy et al. 2008). These evidences imply that high FOXA1 expression is linked with survival and a better outcome in breast cancer patients. Accordingly, a recent publication suggests that FOXA1 directly represses a subset of basal signature genes (Bernardo et al. 2012). In this study, the silencing of FOXA1 causes a partial shift from luminal to basal gene expression signatures, which results in an increased migration and invasion of luminal cancer cells. This phenotype is representative of the basal subtype of tumors, which are negative for ER and HER2 expression. In breast, GATA-3 plays an important role in mammary gland development and differentiation (Bossard and Zaret 1998; Ho and Pai 2007). Moreover, the inactivation of GATA-3 in mice results in contracted mammary epithelial structure, severely impaired lactogenesis, and disrupted differentiation of luminal progenitor cells into ductal and alveolar cells (Asselin-Labat et al. 2007). In breast cancer cell lines, GATA-3 has been positively implicated in mediating the estrogen–ER signaling (Eeckhoute et al. 2007). All in all, FOXA1 and GATA3 that are subsequently used by ER to bind chromatin and regulate gene transcription, respectively (Carroll et al. 2005; Eeckhoute et al. 2007; Hurtado et al. 2011), might be considered as biomarkers of luminal tumors. In fact, 83.1 % of FOXA1-positive tumors are comprised in the luminal A subtype. Similarly, 87.7 % of GATA-3-positive tumors fall within this molecular subtype (Wilson and Giguere 2008; Albergaria et al. 2009).

The pioneer factor FOXA1 also plays an important role in androgen receptor (AR) signaling of molecular apocrine tumors, which have been recently identified as an additional subgroup of ER-negative and AR-positive breast tumors (Ni et al. 2011; Robinson et al. 2011). On the one hand, Ni et al. identified AR as a mediator of the ligand-dependent activation of Wnt and HER2 signaling pathways through direct transcriptional induction of WNT7B and HER3 (Ni et al. 2011). On the other hand, Robinson et al. demonstrated that the specific silencing of FOXA1 inhibits AR binding, expression of the majority of the molecular apocrine gene signature, and cell growth (Robinson et al. 2011). Moreover, Ni et al. proved that specific targeting of AR, Wnt, or HER2 signaling impairs androgen-stimulated tumor cell growth, suggesting potential therapeutic approaches for ER−/HER2+ breast cancers (Ni et al. 2011). Altogether, it seems that, in breast tumors, ER and AR binding and their functionality is fully dependent on FOXA1. By contrast, in prostate cancer, the effect of FOXA1 on AR binding is more complex. Recently, two studies have reported a new paradigm for the forkhead protein FOXA1 action in androgen signaling. Besides the pioneering function on the AR pathway, FOXA1 depletion elicited extensive redistribution of AR-binding sites (Sahu et al. 2011a, b; Wang et al. 2011). Interestingly, both groups identified three distinct classes of AR binding sites and androgen-responsive genes: some independent of FOXA1, others pioneered by FOXA1, and some others masked by FOXA1 and functional upon FOXA1 depletion. Importantly, FOXA1 protein level in primary prostate tumors has a significant association with the disease outcome; high FOXA1 level is associated with poor prognosis, whereas low FOXA1 level, even in the presence of high AR expression, predicts good prognosis. The role of FOXA1 in androgen signaling and prostate cancer (Gerhardt et al. 2012) is different from that in estrogen signaling and breast cancer (Sahu et al. 2011a, b). By contrast, in breast cancer, there is a clear association between high FOXA1 expression and a better survival (Habashy et al. 2008). In fact, the Oncotype DX test for breast cancer prognosis shows a negative and significant correlation between FOXA1 expression and recurrence (Ademuyiwa et al. 2010). In the future, studies focused on these tissue-specific properties of FOXA1 will be instrumental for our understanding of hormone-regulated cancers.

In endometrial cancer tumors, FOXA1 is expressed in 37 % of the cases, and its expression is significantly and negatively associated with lymph node status (Abe et al. 2012). Interestingly, in ER-positive endometrial cancer cells, FOXA1 has been suggested to function as a tumor suppressor through modulation of proliferation and migration of endometrial cancer cells (Abe et al. 2012). However, it is not clear whether FOXA1 action occurs through ER or progesterone receptor (PR). Very recently, Clarke et al. have reported that FOXA1 alters PR transcriptional response in normal breast AB32 cells, a PR-positive clone of the MCF-10A cell line (Clarke and Graham 2012). The conclusions of this study suggest that FOXA1 is not absolutely required for progesterone response. However, when FOXA1 is overexpressed in AB32 cells, it induces the expression of genes involved in negative regulation of apoptosis. Yet, we do not know the role of FOXA1 in progesterone- and estrogen-induced transcription in endometrial tissue.

In summary, all the published data suggest that the idea of using FOXA1 as a therapeutic target in breast and endometrial cancers could be an alternative for those patients with recurrence to current treatments. However, we still have a long way to go. We need to know in what other functions, apart from that of pioneering, FOXA1 is involved. Furthermore, it is important to know how the function of FOXA1 is regulated. Finally, to decipher the specific weight of other pioneering functions that might compensate functionally the inactivation of FOXA1 is critical for future therapies.

## ER-cooperating factors influence estrogen-mediated transcription

**Fig. 2:**
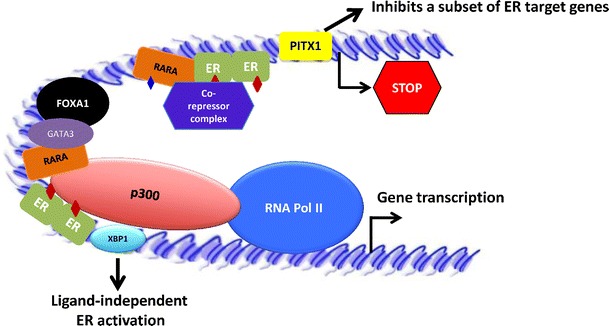
ER-cooperating factors influence estrogen-mediated transcription. In breast carcinoma cell lines, the complex created by FOXA1, GATA3, and ER regulates estrogen (*red bold dot*) transcription. These three factors are necessary for the recruitment of the co-activator p300 and RNA polymerase II. Moreover, XBP1 promotes ER transcriptional activity in a ligand-independent manner. RARA, after binding its ligand ATRA (*blue bold dot*), interacts and cooperates with ER at ER binding sites, where it stabilizes both ER co-activator and co-repressor binding. PITX-1 represses transcription of a subset of ER-regulated genes

Estrogen may activate or repress transcription of ER target genes potentially by recruiting distinct classes of co-regulators that have chromatin remodeling properties. Structural and functional studies revealed that ER co-activators are recruited to hormone-responsive genes through their interaction with activated receptors. In turn, the co-activator complex remodels the chromatin at this region through histone acetylation, facilitating RNA polymerase II-mediated transcription (Onate et al. 1995; Anzick et al. 1997; Torchia et al. 1997; Chen et al. 1999a, b). It has also been established that, in estrogen-repressed genes, estrogen–ER stimulates the selective association of co-repressors (Carroll et al. 2006; Stossi et al. 2009). The interaction of these co-repressors prompts the binding of chromatin deacetylatases and therefore leads to transcriptional inhibition. Some transcription factors have been shown to be responsible for ER cofactor binding (GATA-3, FOXA1, and RARA), to function as cofactors by themselves (XBP1) or to be mediators of ER-repressive action (PITX-1). In the next paragraphs, we discuss the function of these transcription factors (Fig. 2).

Recently, Kong et al. reported in MCF-7 breast carcinoma cells that FOXA1, GATA-3, and ER form a protein complex, which regulates ER-mediated transcription (Kong et al. 2011). The chromatin regions occupied by all these three transcription factors were associated with the highest p300 co-activator recruitment (histone acetylase enzyme), RNA Pol II occupancy, and chromatin opening. Interestingly, co-transfection of these three transcription factors was sufficient to restore the estrogen-responsive growth of ER-negative MDA-MB-231 and BT-549 cells. These findings are very significant and suggest that all three transcription factors are needed for co-activator recruitment and, ultimately, for ER-mediated transcription in breast tissue. However, it is not clear yet whether the complex of ER, FOXA1, and GATA-3 is necessary for all ER-regulated transcripts in breast tissue.

Retinoic acid receptor alpha (RARA) is a nuclear receptor, which regulates gene expression by retinoic acid (RA) (Giguere et al. 1987). Both RA (Darro et al. 1998; Paroni et al. 2012) and antagonists of RARA (Dawson et al. 1995; Toma et al. 1998) promote anti-proliferative effects in breast tumor cells. Moreover, RARA is known to be an estrogen-induced target gene in breast cancer cells (Laganiere et al. 2005a, b). Yet, the mechanisms of action by which the RARA agonists or antagonists carry out a repressive effect in breast cancer are not entirely clear. The White and Carroll groups have tried to solve this conundrum. Although both teams agree that RARA binding throughout the genome is highly coincident with ER binding (Hua et al. 2009; Ross-Innes et al. 2010), they propose contradictory mechanisms of action. Whereas White's group supports a genomic antagonism between RA and estrogen signaling (Hua et al. 2009), Carroll's group supports a cooperative interaction between RARA and ER (Ross-Innes et al. 2010). Altogether, it seems that RARA might have two distinct roles in breast cancer cells: first, repressing estrogen transcription via the classic function of RARA with its interacting partner retinoid X receptor and, second, interacting with ER and maintaining ER–cofactor interaction for estrogen-mediated gene transcription. Given the observation that both RARA agonist and antagonist actions show benefit on breast cancer, both mechanisms of action might be taking place.

XBP1 is a transcription factor that belongs to the basic region/leucine zipper (bZIP) family of proteins (Clauss et al. 1996). Regulation of transcription by XBP1 is a consequence of its binding to and activation of specific cAMP-responsive element. The XBP1 spliced form, XBP1(S), has the ability to bind to and activate ER in a ligand-independent manner (Ding et al. 2003). Furthermore, XBP1 is also rapidly induced in response to estrogen stimulation (Wang et al. 2004; Tozlu et al. 2006; Scriven et al. 2009), which suggests that transcription of ER-regulated genes might be also modulated by the complex ER–XBP1. Recently, the pituitary homeobox 1 (PITX-1) transcription factor has been related as a repressor for a subset of ER target genes (Stender et al. 2010).

All together, these evidences suggest that ER-cooperating factors might be needed for estrogen–ER-mediated transcription. Interestingly, the transcription of some of these cooperating factors is induced both by estrogen at early time points (Hurtado et al. 2011) as directly by FOXA1 (Nakshatri and Badve 2009). This supports the established hypothesis and also suggests that these factors are needed for a sustained transcriptional activity of ER. Perhaps, FOXA1 induces the transcription of these cooperating factors to allow the expression of early ER-regulated genes. Subsequent activation of the transcription of these cooperating factors by ER would then allow the sustained expression of ER targets. However, the mechanism of action of these factors for ER-mediated transcription is not completely understood. It has been suggested that they might be important for recruitment of chromatin remodeling factors (Ross-Innes et al. 2010). Future studies should provide a more comprehensive explanation of the function of these factors.

## ER-cooperating transcription factors and anti-estrogen drug response

Breast tumors positive for ER expression represent around 70 % of these cancers (Dowsett 2001; Prat and Baselga 2008). Targeting estrogen action has been a therapeutic choice of breast cancer treatment so far (Harvey et al. 1999). In the last 30 years, various endocrine treatments have been developed in order to block estrogen action in breast cancer cells. One of the most successful treatments is represented by the SERM tamoxifen (Jensen and Jordan 2003). It antagonizes estrogen action by competing for the binding of ER in breast cancer cells and is thought to repress ER-mediated transcriptional activation by actively recruiting co-repressors (Katzenellenbogen and Frasor 2004; Malik et al. 2010; Hurtado et al. 2011). More recently, another class of anti-estrogen drug has been incorporated into clinical treatment, namely aromatase inhibitors (AI). These inhibitors antagonize estrogen metabolism, and therefore, their use is restricted to postmenopausal women. Unfortunately, one third of women treated with any of these treatments will relapse (Musgrove and Sutherland 2009). The molecular mechanisms by which tamoxifen induces repression on breast cancer cells are not completely understood (Harvey et al. 1999), and diverse models of endocrine resistance have been hypothesized (Higgins and Stearns 2009). In this section of the review, we discuss which transcriptional cooperating factors can affect ER genomic activity in response to endocrine treatment and how this process can affect cell proliferation and survival.

The effectiveness of tamoxifen requires both the binding with ER and the consequent interaction with DNA. Importantly, genomic maps of ER binding induced with estrogen or tamoxifen are almost identical (Hurtado et al. 2011), which evidences that tamoxifen–ER uses the same genomic regions as estrogen–ER for its repressive action. From these evidences, one might assume that tamoxifen–ER and estrogen–ER use the same mechanisms to interact with DNA (Hurtado et al. 2011). In agreement, the expression of FOXA1 is essential for ER–tamoxifen inhibitory action. Interestingly, Ross-Innes et al. observed that, in tumors resistant to endocrine therapy, ER interactions were enriched with FOXA1 motifs (Ross-Innes et al. 2012). From these studies, one can get the conclusion that FOXA1 is needed to permit ER–tamoxifen interaction with DNA but is not sufficient to induce repression.

**Fig. 3:**
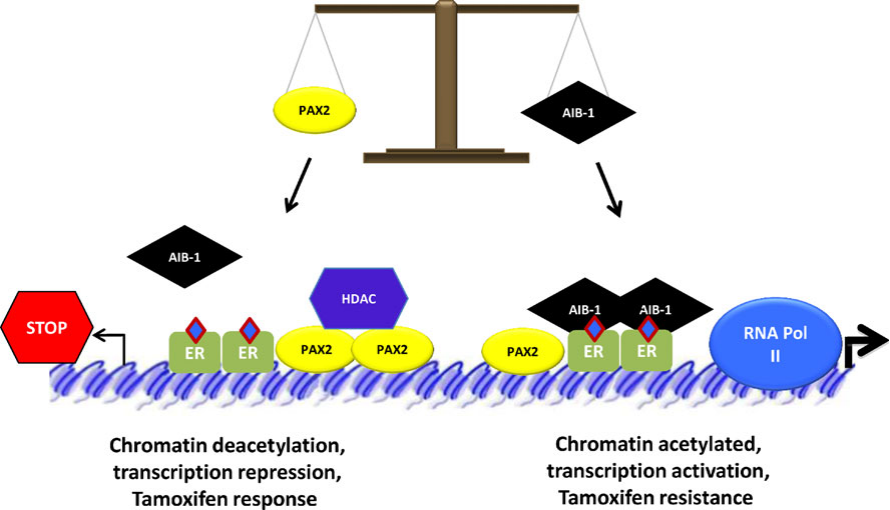
The balance between AIB-1 and PAX2 governs ER–tamoxifen action. In breast cancer cells, after tamoxifen treatment (in *blue*, bound to ER), PAX2 and AIB-1 compete for the binding of ER, and this competition determines tamoxifen response. High levels of PAX2 may recruit co-repressors and other factors that promote chromatin compaction, ER-mediated repression, and tamoxifen sensitivity (on the *left*). On the contrary, high levels of AIB-1 promote chromatin opening, transcription activation, and tamoxifen resistance (on the *right*)

Crosstalk between the ER and HER2 pathways has long been implicated in cancer onset and response to tamoxifen, but no direct connection at transcriptional level has been shown. Tamoxifen-resistant breast tumors are characterized by elevated *ERBB2* levels (Osborne et al. 2003), and ER-positive cell line models overexpressing *ERBB2* acquire resistance to tamoxifen (Benz et al. 1992). In 2008, a new mechanism of resistance to tamoxifen treatment was suggested (Hurtado et al. 2008), which revealed a novel interplay between ER and *ERBB2* on a genomic level. This study proposes that the anti-proliferative effects of tamoxifen require repression of *ERBB2* and that breast cancer cells acquire resistance by deregulating the mechanisms that normally repress *ERBB2* transcription. This repression is mediated by the transcription factor PAX2, which cooperates with ER at this locus. PAX2 belongs to the pair box gene (PAX) family, a group of transcription factors characterized by the presence of two DNA-binding domains and are known for their role in terminal differentiation during organogenesis (Mansouri et al. 1996; Dahl et al. 1997). PAX2 is expressed in around 60 % of breast tumors (Muratovska et al. 2003), and its nuclear localization is more frequent in luminal tumors than in nonluminal tumors (Silberstein et al. 2002; Liu et al. 2009). Moreover, in luminal breast cancer cell lines, PAX2 has been shown to be activated and confers a low invasive phenotype (Beauchemin et al. 2011). Interestingly, PAX2 silencing is able to abrogate the inhibition of *ERBB2* transcription and increases *ERBB2*-dependent cell proliferation (Hurtado et al. 2008). Moreover, the expression of PAX2 is reduced in tamoxifen-resistant cells, and the overexpression of PAX2 is able to restore the sensitivity to tamoxifen in these cells (Hurtado et al. 2008). Nonetheless, another study showed that changes in *ERBB2* expression are not dependent on differences in PAX2 expression among various populations of tamoxifen-resistant and estrogen-deprived MCF-7 cells (Leung et al. 2010). Tamoxifen-resistant cells are also characterized by increased levels of the ER co-activator AIB-1 (Osborne et al. 2003; Su et al. 2008). Indeed, there is a competition between AIB-1 and PAX2 for the binding to the ER binding region of the *ERBB2* gene, which might explain the differences between the two studies. This event has also been shown in tamoxifen-treated breast cancer samples, where the PAX2-positive AIB-1-negative tumors have the best prognosis and the lowest percentage of *ERBB2*-positive cells (Hurtado et al. 2008). These results suggest that the critical event for *ERBB2* repression and tamoxifen resistance is not just explained by the loss of PAX2 expression, but it supports the idea that the balance between PAX2 and AIB-1 recruitment at chromatin level is crucial for the determination of tamoxifen response and resistance. Yet, the molecular mechanism underlying the competition between PAX2 and AIB-1 in ER-mediated regulation of transcription is not completely understood. We have observed that tamoxifen mainly enhances the binding of PAX2 at genome-wide level (Gilfillan et al. 2012), which suggests that PAX2 might be functioning as a general repressor for ER–tamoxifen action (Fig. 3). However, all the genomic regions where PAX2 interacts with DNA after estrogen or tamoxifen treatment have not been identified yet, and therefore, it cannot be established whether PAX2 is required for all estrogen-repressed genes. Furthermore, it is not clear yet if the competition between PAX2 and AIB-1 might be affecting the transcriptional regulation of many different ER target genes. In summary, all these studies denote that the repressive action of tamoxifen is regulated by cooperating factors at least at two different levels: FoxA1, which orchestrates ER binding on the chromatin, and PAX2, which dictates the transcriptional activity of ER induced by tamoxifen. Furthermore, all these findings highlight the complexity of ER–tamoxifen transcriptional regulation in human breast cancer.

Although tamoxifen is a successful ER antagonist in breast cancer therapy, it shows partial agonistic effects in other target tissues (Fisher et al. 1998; Jordan et al. 2001). In particular, tamoxifen treatment has been associated with an increased incidence of endometrial carcinoma (Persson 2000; Zeleniuch-Jacquotte et al. 2001). Gene expression analysis has shown that the genes targeted by tamoxifen are different from those targeted by estrogen, in endometrial epithelial cancer cells (Wu et al. 2005). PAX2 is a common target of estrogen- and tamoxifen-bound ER and is a crucial effector for the proliferation of endometrial cancer cells. Furthermore, PAX2 is silenced in normal endometrium, and its expression is reactivated in endometrial cancer upon hypomethylation of its promoter. Altogether, these evidences suggest that PAX2 plays a crucial role in the determination of tamoxifen response both in breast and endometrial cancer cells, by repressing and promoting cell proliferation, respectively. For these reasons, further studies on the role of PAX2 in cooperation with ER may shed light on tamoxifen molecular mechanisms of action and resistance.

**Fig. 4:**
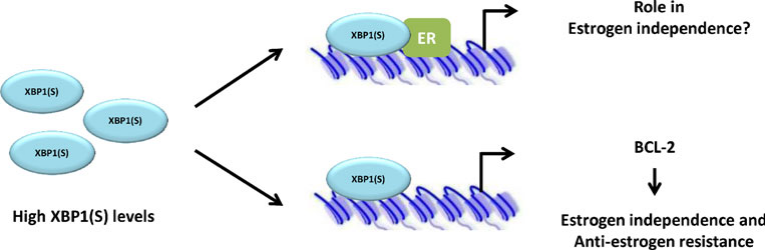
XBP-1(S) has a role in ligand-independent ER activation and anti-estrogen drug resistance. XBP-1(S) overexpression plays a dual role in estrogen independence and anti-estrogen resistance. XBP-1(S) can bind and activate ER in a ligand-independent manner (*upper panel*) and induces transcription of BCL-2 gene (*lower panel*), which might have implications in anti-estrogen drug resistance

In previous sections, we have discussed the role of XBP-1(S) in ligand-independent activation of ER. Moreover, XBP-1(S) is also a key mediator of ER-independent growth (Gomez et al. 2007; Davies et al. 2008). Gomez et al. showed that just the overexpression of XBP-1(S) explained both phenotypes (Gomez et al. 2007). Importantly, the study confirms XBP-1(S) as an essential regulator of BCL2 transcription, which is a prosurvival/antiapoptotic factor and confers resistance to aromatase therapy in breast cancer patients (Ding et al. 2003) (Fig. 4). For these reasons, XBP-1(S) may be considered an important diagnostic and prognostic biomarker of breast cancer samples and may be also a useful tool in the identification of ER-positive breast tumors with a relatively poor response.

## Cell signaling pathways modulating ER and ER-cooperating factors

**Fig. 5:**
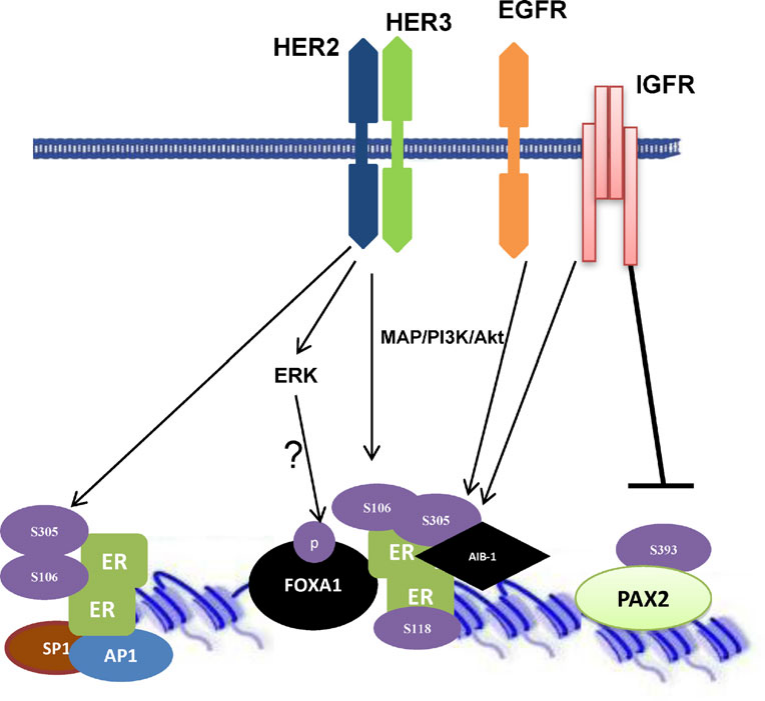
The crosstalk between growth factor signaling pathways and ER-cooperating factors. Receptor tyrosine kinases EGFR, HER2, and IGFR1 activate downstream signaling pathways including PI3K/Akt, MAP kinases, and ERK. These kinases may phosphorylate ER, which can be activated in a ligand-independent manner. HER2 signaling also regulates FOXA1. ER is activated and interacts with other transcription factors to bind chromatin. IGFR-1 represses PAX2 transcription factor by inducing specific phosphorylation

In addition to ligand binding, posttranslational modifications (acetylation and phosphorylation) of ER and its associated co-activators (e.g., SRC1, SRC2, AIB1, p300) and co-repressors (e.g., MTA1, NCoR, and SMRT) play a role in ER action. Histone-modifying enzymes interact with ER and influence its activity and that of its cooperating factors. Yet, how the recruitment of these enzymes is regulated is an open question. Moreover, in response to estrogen treatment, ER can activate a variety of kinases (e.g., MAP kinases, ERK, and AKT) and phosphatases (e.g., PP1, PP2A, and PDXP), which can regulate histone proteins (e.g., Msk1, Msk2, and histone H1) and ER co-regulators. In this section, we review and discuss the importance of these enzymes in modulating ER, ER cooperating partners, and their relevance in hormone-resistant tumors (Fig. 5).

ER is modulated by membrane receptor tyrosine kinases/growth factor signaling, including epidermal growth factor receptor (EGFR), HER2, and insulin-like growth factor receptor (Nicholson et al. 2002; Schiff et al. 2003), which contributes to endocrine resistance (Drury et al. 2011; Fagan et al. 2012). The overexpression of these receptor kinases can activate the downstream MAPK/ERK and PI3K/AKT pathways (Kato et al. 1995; Chen et al. 1999a, b; Rayala et al. 2006; Miller et al. 2011), which results in phosphorylation of ER at multiple serine residues (e.g., 104, 106, 118, 167, and 305), and can influence ER signaling (Arnold et al. 1994; Chen et al. 2002; Likhite et al. 2006; Thomas et al. 2008; Williams et al. 2009). Importantly, phosphorylation of ER at serine 305 is associated with endocrine resistance and poor prognosis (Kok et al. 2011, Houtman et al. 2012). The phosphorylation of ER by cytoplasmic kinases also regulates its function via interaction with other transcription factors such as AP-1, SP1, and CREB, which mediate ER interaction with chromatin (Porter et al. 1997; Kushner et al. 2000; Zhou et al. 2005).

HER2 signaling has also connection with the ER-cooperating factors FOXA1, PAX2, and AIB-1. In molecular apocrine breast tumors, HER2 regulates FOXA1 via ERK phosphorylation (Naderi et al. 2012). However, the precise mechanism behind the crosstalk between HER2 and FOXA1 signaling is not completely understood yet. HER2 signaling also induces AIB-1 phosphorylation (Osborne and Schiff 2003), which contributes to tamoxifen resistance. Moreover, IGF-1 negatively regulates PAX2 activity in breast by inducing its phosphorylation (Beauchemin et al. 2011). Perhaps, regulation of protein phosphorylation might be a mechanism of control of the activity of PAX2 and AIB-1 and may ultimately dictate the outcome to anti-estrogen therapies.

In summary, ER signaling and its crosstalk with various signaling pathways have been clinically associated with poor clinical outcome and resistance to anti-estrogen therapies. Therefore, affecting either kinases or phosphatases regulating ER might help in treating patients with resistance to these therapies. Importantly, PP1 phosphatase is known to dephosphorylate AIB-1, and this results in suppression of its degradation (Li et al. 2008). The other phosphatases PDXP and PP2A inhibit SRC3 interaction with ER in the absence of ligand (Li et al. 2008). Future studies identifying how these phosphatases and kinases regulate ER and its cooperating factors might improve the anti-estrogen therapies.
